# Editorial Comment from Dr. Mori to texture feature analysis using dynamic computed tomography for preoperative risk stratification in upper urinary tract urothelial carcinoma

**DOI:** 10.1111/iju.15291

**Published:** 2023-08-28

**Authors:** Keiichiro Mori

**Affiliations:** ^1^ Department of Urology The Jikei University School of Medicine Tokyo Japan; ^2^ Department of Urology Medical University of Vienna Vienna Austria

Upper tract urothelial carcinoma (UTUC) is a relatively rare malignancy that accounts for 5%–10% of all urothelial carcinomas.^1^ To improve oncological outcomes in patients with UTUC, preoperative predictive models have been developed to guide clinical decision‐making and patient counseling.^2^ However, preoperative risk assessment for UTUC has not been fully established.^3^

In this issue of the *International Journal of Urology*, Kaneko et al. aimed to assess the utility of texture analysis using preoperative computed tomography (CT) images for risk stratification of UTUC.^4^ They concluded that texture analysis using preoperative dynamic CT images may identify one useful risk factor related to metastatic recurrence following radical nephroureterectomy. CT urography (CTU) has become the new gold standard for this type of tumor detection.^5^ However, despite the availability of advanced imaging technologies, it remains difficult to achieve sufficient staging accuracy to ensure a tailored treatment strategy for patients with UTUC. Additionally, CTU appears to have limited value in terms of providing accurate information regarding tumor stage and grade. Thus, reliable preoperative prognostic factors are of particular interest, given that patients at high risk of tumor progression may benefit from preoperative chemotherapy and lymphadenectomy. In this regard, this is an important topic and a valuable paper. Kaneko et al.'s study provides a good opportunity to discuss the utility of texture analysis using preoperative CT images for risk stratification of UTUC.

However, there are a few key limitations to this study that are also worth noting. First, multivariable analysis to investigate the impacts of texture features on recurrence was not performed, due to the limited number of events studied. Second, the limited number of cases did not allow for further analyses regarding a possibly different impact of the texture features according to primary lesions. Our interest lies in whether there are any differences in terms of the prognostic impacts of texture features between renal pelvis and ureter tumors. Thus, future studies are needed to address a possible difference in the prognostic impact of texture features between renal pelvic cancer and ureteral cancer.

## CONFLICT OF INTEREST STATEMENT

The author has nothing to declare.
